# Comparative Study of the Composition of Sweat from Eccrine and Apocrine Sweat Glands during Exercise and in Heat

**DOI:** 10.3390/ijerph17103377

**Published:** 2020-05-12

**Authors:** Yi-Lang Chen, Wen-Hui Kuan, Chao-Lin Liu

**Affiliations:** 1 Department of Industrial Engineering and Management, Ming Chi University of Technology, New Taipei 24301, Taiwan; ylchen@mail.mcut.edu.tw; 2 Department of Industrial Design, Chang Gung University, Taoyuan 33302, Taiwan; 3 Department of Safety, Health and Environmental Engineering, Ming Chi University of Technology, New Taipei 24301, Taiwan; 4 Chronic Diseases and Health Promotion Research Center, Chang Gung University of Science and Technology, Chiayi 61363, Taiwan; 5 Department of Chemical Engineering, Ming Chi University of Technology, New Taipei 24301, Taiwan; clliu@mail.mcut.edu.tw; 6 Department of Chemical and Materials Engineering, Chang Gung University, Taoyuan 33302, Taiwan

**Keywords:** analysis of variance (ANOVA), apocrine sweat gland, dynamic exercise, eccrine sweat gland, heat environment

## Abstract

This preliminarily study was made to examine the differences in sweat excretions from human eccrine and apocrine sweat glands in dynamic exercise and heat conditions. Sweat samples were collected from six young males while they were either running on a treadmill or sitting in a sauna cabinet. Sweat samples of at least 5 mL from the eccrine (upper^−^back) and apocrine (armpit) sweat glands were collected during a 20^−^min running (or inactive overheating) period. The samples were then analyzed for urea, uric acid, and electrolyte (Na^+^, Cl^−^, and K^+^) excretions. The results from a two^−^way repeated^−^measures analysis of variance (ANOVA) revealed that the secretions of urea and K^+^ were significantly higher during running than during inactive overheating for both glands, as were Na^+^ secretions for the apocrine glands (all P < 0.05). Under the same sweating conditions, urea and K^+^ excretions from the apocrine glands were also higher than those from the eccrine glands (all P < 0.05). Significant differences were observed between the Na^+^ secretions of the apocrine and eccrine glands under the running condition. The effects of various sweating methods and sweat glands on Cl^−^ secretions were nonsignificant, and little uric acid was excreted. A higher urea excretion level during running rather than in hot conditions could be attributed to an elevated metabolic rate.

## 1. Introduction

Perspiration is an essential physiological function for hydration and the regulation of both hemostasis in electrolytes and body temperature through sweat ducts in the skin. Detoxication is also a function of sweating that occurs through the excretion of harmful molecules, such as urea [1,2]. Human skin, like the kidneys, controls and regulates water output and directs the secretion of the key electrolytes Na^+^ and Cl^−^ through resorption in the ducts. When humans exert themselves, thermal stasis is maintained and toxic metabolic products are removed through both perspiration and up-regulation of blood flow. The metabolic rate is also increased and metabolite disposal, a pathway for body metabolic clearing, is promoted. Therefore, sweating usually accompanies exercise and may contribute to cardiovascular disease prevention [3,4,5,6]. Furthermore, the relative concentrations of unmetabolized drugs are occasionally higher in sweat than in blood, urine, or saliva [7,8]. This indicates that some components of sweat are human physiological and pathological indicators.

Up to 30% of bodily waste is secreted through perspiration from the sweat glands of over 2.6 million tiny pores in the human skin. These glands are categorized as either eccrine or apocrine [1,9]. Although a third type of sweat gland was identified by Sato and Sato in 1987, most relevant literature generally refers to either eccrine or apocrine sweat glands [10,11]. Eccrine sweat glands are present at birth and cover the entire surface of the body, whereas apocrine glands are dormant until puberty. The distribution of apocrine glands is restricted in hairy parts of the axilla, mammary, perineal, and genital regions. The composition of sweat from the two gland types also differs. The components of eccrine are dependent on diet, hydration, metabolic rate, state of health, drug administration, and body area [12]. These components are composed primarily of water but also small amounts of minerals (such as sodium, potassium, calcium, and magnesium), metabolites (such as lactate, ammonia, and urea), and unmetabolized pharmaceutical drugs. By contrast, the fluid secreted from the apocrine sweat gland is an oily, odorless substance consisting of proteins, lipids, and steroids, but contains same minerals and metabolites. However, studies on sweat composition have predominantly focused on sweat from the eccrine sweat glands. Quantitative research evaluating the differences between eccrine and apocrine sweat glands with regard to metabolites and electrolytes is scant. Such research would be practically valuable if the differences could be empirically identified.

Furthermore, few studies have focused on the conditions influencing sweat secretion as its components. Baker et al. [13] established the normative data for regional sweat Na^+^ concentration and whole^−^body sweating rate in athletes. Verde et al. [14] examined sweat composition under exercise and heat conditions and revealed that Na^+^, Cl^−^, and K^+^ contents were similar for outdoor exercise in a cool environment, indoor exercise at a normal room temperature, and exposure to a steam sauna. However, because the humidity level was almost saturated in the sauna exposure, the collected sweat samples may have been confounded. Other researchers have examined the composition of sweat secreted during exercising in the sun [15], in hot environments [2], in an air^−^conditioned room [16], and in a hot chamber [17]. However, few comparative studies have been conducted. Consolazio et al. [18] performed a comparative study and indicated that the sweat calcium accounted for 20.6% of the daily total body loss in a temperate environment, 36.7% in both exercise and hot environments, and 22.7% in a hot environment without exercise. These results might indirectly imply that exercise led to more calcium excretion than hot conditions alone did, although they did not assess sweat secretion levels during exercise without heat. Hussain et al. [19] also suggested because this is still an active research area, it cannot be assumed that metabolomics studies in which exercise and/or inactive overheating were employed to induce sweating have interchangeable results. Reports on the composition of human sweat have generally been inconsistent because of variations in parameters [1,14]. The types of conditions included in the experiment (exercise, heat exposure, or a combination of the two stimuli), duration of sweating, rate of sweat secretion, and methods of sample collection are all variables. The experimental setting should therefore be considered when interpreting sweat^−^composition data. The present study therefore attempted to examine the differences in sweat composition (urea, uric acid, Na^+^, Cl^−^, and K^+^) between eccrine and apocrine sweat glands under conditions of exercise and heat, respectively. This can help to inform what sweating stimulus should be adopted for different studying purposes and elucidate how sweating can contribute to the maintenance of personal hygiene [20] and the optimal textile design of sport gear [21].

## 2. Methods

### 2.1. Participants

Six young, male university students were recruited as participants; the mean (standard deviation) age, height, and body mass of participants were 22.2 (0.4) years, 171.9 (7.8) cm, and 68.9 (8.6) kg, respectively (Table 1). All participants reported being moderately physically active (leisurely exercise at least twice a week), healthy and asymptomatic of illness and no pre^−^existing injuries. Table 1 also shows the maximal peak expiratory flow among the participants at a similar level. The maximal peak expiratory flow was measured using a Wright peak flow mini^−^meter (Clement Clarke, 3103001, Essex, UK). The maximal flow is limited by the rate by which the muscles are able to transform chemical energy into mechanical energy and also by a rising flow resistance, it is reduced in persons who have any airway obstructions [5]. In the previous year, all participants dined in the same restaurant; for one week before and during the experiment, their food intake was controlled so that they all ate the same, thus minimizing any biases resulting from variation in daily individual food intake. Furthermore, no medication was allowed for all participants during the test, otherwise they will be excluded from the test. The experimental procedures were approved by the Ethics Committee of Chang Gung Memorial Hospital, Taiwan (ethical code: No. 983653A3) and all study participants provided written consent prior to the experiment.

### 2.2. Stimulated Sweating and Environmental Conditions

This study involved two stimulated sweating conditions: running (on a treadmill; CS^−^5728, Chanson, Taipei) and inactive overheating (in a traditional heat sauna cabinet). To assess the influence of sweat rate on the composition of sweat produced under various conditions (running and heat in this study), the sweat rate was measured and used for a pilot test. The temperature of the running room and the sauna cabinet were set at 25 °C and 45 °C, respectively, and the humidity was set at the same level (40%). The temperature range of the sauna cabinet was 40 °C to 60 °C, with 5 °C increments. Identical humidity levels were also implemented to control sweat evaporation. The humidity of the environment in the study was considered a controlled variable to eliminate differences in sweat evaporation between the two sweating conditions.

During the running test, participants were requested to run on a treadmill, the speed of which increased gradually from 5 to 10 km/h during the first 10^−^min, followed by a further 10 min of running. For the heat condition, sweat tests were performed in a sauna cabinet for 20 min. During the sweat^−^collection period, sweat from the upper^−^back (eccrine) and armpit (apocrine) regions [1] of participants was collected until 5 mL of sweat had successfully been obtained from each region.

### 2.3. Sweat Collection

Previous studies employed a diverse range of methods to collect sweat for composition analyses [19,22,23]. These methods include the simple and direct collection of sweat from the skin using tubes or glass jars, specifically designed implementations (e.g., glass pipettes and rollers, hydrogel micropatches, arm bags, and sweat pouches), and commercial products. Skin irritation, pH alteration, disturbance of barrier properties, and variations in location at which sweat is produced and the purpose of examination lead to difficulties in designing a universal and ideal sweat^−^collection apparatus [24].

This study attempted to both understand the composition of sweat under conditions that were as natural as possible and develop a sweat collector capable of collecting sufficient volumes of sweat with minimal skin interference for the required duration. Other studies have employed direct sweat^−^collection methods using tubes or pipettes [25,26,27,28,29,30]. In this study, however, the homemade sweat collector comprised a funnel with a glass tube, which was cleaned using deionized water, air^−^dried, and then covered with tin foil to prevent contamination. Collectors capable of holding different volumes were prepared and coded prior to the tests in accordance with the volume of the samples required. When collecting sweat, the experimenter used the funnel edge to separately scrape the sweat from the upper^−^back and armpit regions of participants, which were selected with reference to the study conducted by Wilke et al. [1]. The pilot tests ensured that the level of sweat^−^sample contamination caused by the collector and procedure was minimal. The composition of the obtained target sweat was satisfactory and reproducible. The required sweat volumes and sweating rates for the sampled regions were also examined and confirmed through pilot tests.

### 2.4. Experimental Design and Procedure

During the test, the sweat produced under different conditions was collected from the target sample regions (8 cm × 8 cm) using the homemade sweat collector within a 20 min running (or inactive overheating) period and an extended sampling standing period. After several pilot runs, an additional 30^−^min collection extension was used to collect a sufficient volume (>5 mL) of sweat for further chemical analysis. The sweat rates of two body regions under two sweating conditions were determined using the weighing method, and were nearly equivalent, at approximately 1.8 mg/cm^2^/min during the 20 min running (or inactive overheating) and additional 30 min collection extension in standing. Subsequently, a sweat sample of 5 mL was separately collected from the upper^−^back and armpit regions of participants during each normal 50 min course.

Prior to testing, all participants were asked to control their diet and exercise for the duration of one week. Two hours before the test, the participants were asked to urinate, after which they began fasting and were allowed a water intake of only 200 mL. Before sweating began, the experimenter first cleaned the sweat^−^producing regions of the body (upper^−^back and armpit) using a brush and a large amount of tap water to remove any dirt and skin residue, such as dander and grease. Deionized water was then used to clean the skin regions that had been wiped to ensure that they were clean. Ely et al. [31] reported that when the arm^−^bag technique was employed, the skin surface was contaminated by skin desquamation and trace mineral residues (iron, zinc, copper, magnesium, and calcium) because the removal of dirt and other minerals under the finger nails was difficult, even with a meticulous cleaning process. When conducting the same cleaning procedure, the trace mineral concentrations of the upper^−^back sweat sample remained unchanged during 3 h of exercise. Because the sweat sampling areas in this study were easier to clean, a rigorous cleaning process was conducted to minimize possible skin contamination. Consequently, a total of 24 sweat samples were collected (6 participants × 2 sweating environments × 2 sweating regions). A counterbalanced method was employed in the experiment, whereby all participants were randomly divided into either group A or group B. Group A performed the running test first and then the heating test, whereas group B performed the tests in the opposite order. The tests were separated by a 2^−^day interval to prevent cumulative fatigue.

### 2.5. Sweat Preservation and Composition Analysis

At the end of each trial, sweat samples were immediately filtered using a syringe filter with pores measuring 0.22 μm in diameter to remove any cellular debris. The filtering process was conducted to protect the chemical analytical instruments because small particles in liquid samples may have become trapped in the column during the ion chromatography (IC) or during pulverization for inductively coupled plasma^−^atomic emission spectroscopy (ICP^−^AES). Nevertheless, the filter pore size (0.22 μm) was smaller than the skin flakes reported by Mackintosh et al. [32] to be larger than 10 μm. The filtrate was then collected in a capped glass tube and stored at 4 °C. All composition analyses were completed within 32 h following the start of each trial. The sweat samples were analyzed for concentrations of Cl^−^, Na^+^, K^+^, urea, and uric acid. The Cl^−^ concentration was analyzed using IC (Dionex, ICS^−^1500) with a guard, analytical columns (Dionex, AS4A^−^SC 4 mm), a suppressor device, and a conductivity detector. Analyses of Na^+^ and K^+^ were performed using ICP^−^AES (Perkin Elmer, Optima 2100DV). Urea and uric acid were measured using the established method of Fuji dry chemistry, which involved briefly dropping 10 μL of the filtered sweat sample into the dry slide reagent and then measuring the concentration using a dry chemistry analyzer (Dri^−^chem Fuji, NX500).

### 2.6. Statistical Analysis

This study recruited six healthy, male participants and compared five sweat compositions from two sweat glands (eccrine vs. apocrine sweat glands, located in the upper^−^back and armpit) under two sweating conditions (running vs. inactive overheating). The assessment of sweat compositions involved measuring levels of urea, uric acid, and electrolytes (Na^+^, Cl^−^, and K^+^). A two way repeated^−^measures analysis of variance (ANOVA) was used to investigate the effects of each independent variable on sweat composition, and the paired comparison was also employed. Statistical analyses were performed using the statistical software package Statistical Product and Service Solutions (SPSS, Version 21.0). An alpha of 0.05 was used to indicate the minimum level of significance.

## 3. Results

In the analyses, the samples were collected as described from different body regions for the two types of sweat glands [1]. The components of sweat were determined and analyzed using an ANOVA and the post^−^hoc test. As displayed in Table 2, urea, Na^+^, and K^+^ excretions were significantly affected by both sweating conditions and sweat glands (all *P* < 0.05); however, all interaction terms were nonsignificant (all *P* > 0.05). By contrast, Cl^−^ excretion did not differ across the sweat variables. Uric acid excretion was trace (<0.3 mg/dL) and therefore excluded from the analysis.

**Figure 1:**
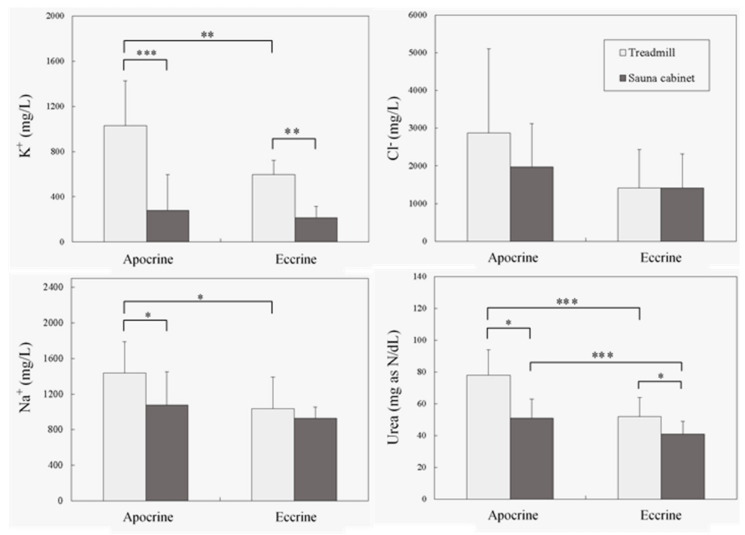
Mean (standard deviation) urea and electrolyte excretions under different test conditions and the paired comparison results (* P < 0.05, ** P < 0.01, *** P < 0.001).

Figure 1 further illustrates the mean (standard deviation) excretions of sweat compositions and the corresponding results of paired comparisons obtained using the post^−^hoc tests. Excretions of urea, the primary metabolic waste, differed significantly in the four paired comparisons. Urea levels were much higher from apocrine glands during running, which suggested that urea is efficiently removed by apocrine glands during active perspiration. Of the ions, Na^+^ and K^+^ excretions were significantly affected by both sweating conditions and sweat glands, whereas Cl^−^ excretion did not differ between the various sweat variables. The measured K^+^ excretion produced by the apocrine sweat glands during running (1029 mg/L) was 4.76 times higher than that measured in the eccrine glands during inactive overheating (216 mg/L). Under the same conditions, the excretions of urea and Na^+^ also increased by as much as 1.90 (78 mg as N/dL/41 mg as N/dL) and 1.55 (1438 mg/L/927 mg/L) times, respectively. In summary, excretions of both Na^+^ and K^+^ only differed significantly between the two glands during running on a treadmill. A divergence was also observed between the two conditions in the two glands for K^+^, but the difference between the two conditions was only significant for Na^+^ collected from the apocrine gland. These results indicated that higher levels of urea, Na^+^, and K^+^ excretion were produced from running than from inactive overheating, the implication being that the excretion of urea and cations could be promoted by the apocrine gland during exercise.

## 4. Discussion

An analysis revealed higher excretion levels of urea, Na^+^, and K^+^ during running than in inactive overheating and in the apocrine than in the eccrine sweat glands. Urea is generally regarded as one of the primary metabolic products evident in human perspiration (along with other chemical compositions, such as ammonia, lactic acid, or heavy metals) and accounts for approximately 1% of the overall composition of sweat [33]. During the process of being eliminated from the blood, urea can pass through the glandular wall and the cell membranes of sweat glands [11,34]. Moreover, its concentration in sweat is generally higher than that of plasma [6,35].

The higher levels of urea excretion in the running group may be a result of more urea having been synthesized. The energy used during bodily movement invokes the operation of the alanine^−^glucose cycle to facilitate gluconeogenesis in the liver. An elevated metabolic rate may be the reason exercise is beneficial to the human body. Thus, the conditions under which sweating occurs should be considered because inherent differences in urea excretion may exist between dynamic and static sweating conditions. Dynamic exercise (i.e., running) may therefore be a more effective means of expelling urea from the body.

The higher concentration of urea and ions identified in secretions from the apocrine sweat glands may result from inherent differences in the mechanisms and physiologies of different sweat glands [9]. Apocrine glands secrete by pinching off outer cell parts; thus, hydrophobic substances, such as lipid derivatives, can be expelled along with the liquid. By contrast, eccrine glands only secrete the liquid from the cell [1]. Because of cell disintegration in the apocrine glands, the secretion is not diluted. The cell disintegration rate may be elevated as a result of the increase in metabolic rate induced by exercise, leading to the higher concentration of molecules in the running group than in the inactive overheating group.

Studies have reported potassium concentrations of 10–5 mM in the arm bag during walking (1.56 m/s, 3.0%), 3.0–10 mM in the dry sauna cabin using the whole^−^body method, and 18–22 mM on the hand/palm [30,36,37]. The potassium concentrations reported in the current study are consistent with those of the aforementioned studies. However, the potassium concentration in sweat is usually independent of the sweat flow rate and not related to that in the blood [38,39]. Moreover, the mechanism remains unclear, and the reabsorption of potassium in sweat may be inactive. High electrolyte concentrations have been hypothesized to be the result of electrolyte leaching from the stratum corneum of the skin [40]. Some ions present in sweat and blood, such as Na^+^ and Cl^−^, are also discrepant [16]. Both are passively reabsorbed through the channel or regulator [41,42,43], resulting in lower concentrations in sweat than in blood.

The higher excretion levels of Na^+^ and K^+^ during running could be attributed to muscle contractions and heat production [5,44]. Muscle contractions are triggered by electrical impulses from nerves. During this process, K^+^, Na^+^, and other compositions function as electrolytes, meaning that they balance the amount of fluid in the body and transmit minute electrical nerve impulses. Na^+^ and K^+^ are essential not only to the nervous system but also to muscle contraction. In this study, dynamic exercise may have required more interactive depolarization and repolarization processes to induce muscle contractions than did static heating, resulting in a greater use of Na^+^ and K^+^. Although the human body minimizes electrolyte loss during exercise, higher levels of Na^+^ and K^+^ excretion may be caused by an alteration to the Na^+−^K^+^ balance as a consequence of modifications to the muscle^−^cell membrane [45]. The concentration of Na^+^ has also been positively associated with sweat rate [46]. During exercise, the core body temperature increases because the excess heat produced, when combined with muscle contractions, increases the sweat rate to promote Na^+^ excretion. Therefore, higher levels of electrolytes can be measured in the sweat produced during exercise than in that produced by inactive subjects in a hot environment. A similar study conducted by Fukumoto et al. [47] employed different collection methods and exercise intensities to examine the sweat excretions from the eccrine glands (i.e., anterior chest), thus caused the results somewhat different from this study. Even though the studies may vary in experimental settings based on different studying purposes, sodium restriction is generally advised for prevention from cardiovascular diseases. For this purpose, diet therapy and exercise is recommended. The data presented in this study also strongly supported this viewpoint because more sodium is intracorporally excreted by the external route of sweating than by urinary excretion [47].

Cl^−^ excretion in this study yielded nonsignificant results (Table 2), irrespective of the environment or gland type. As shown in Figure 1, a difference in Cl^−^ excretion appeared to exist between the two sweating environments; however, this effect was eclipsed by individual differences, which may be due to the relatively small sample size used in the study and merits further investigation. Regarding the lower uric acid metabolic rate revealed in the analysis, this may be because the participants recruited in the test were healthy, young men who exercised regularly; therefore, the high levels of uric acid obtained can be easily attributed this fact [34].

The results also reveal that with the exceptions of uric acid and Cl^−^, higher excretions of urea, Na^+^, and K^+^ were released in the apocrine glands than in the eccrine glands. This phenomenon was more evident when participants performed the running test, implying that the composition of sweat differs according to the body region and type of sweat gland producing it. Another notable finding of this study was that higher urea, Na^+^, and K^+^ excretions measured in the apocrine glands also endorsed the popular opinion that when showering, people should “…minimize soaping to only the oily and odor^−^causing parts of your skin such as your face, armpits, buttocks, groin, and feet” [20]. Sex hormones are generally believed to have a major effect on apocrine gland maturation and activation [1]. The higher sweat excretions (e.g., urea) from the apocrine glands after running reported in this study may lead to more chemical reactions with the air, thus resulting in more intense odors.

One major limitation of this study was that the upper^−^back and armpit sites were assumed as the typical eccrine and apocrine sweat glands in the study, respectively. It may be more accurate to describe the sweat collected in this study simply as ‘upper back sweat’ and ‘armpit sweat’ representing systems biology^−^based fluid collections with multiple sources – ‘upper back sweat’ comprising eccrine and sebaceous sweat gland secretions as well as surface skin cellular debris and skin microbiota^−^influenced metabolites and ‘armpit sweat’ likewise comprising eccrine, apocrine, and apoeccrine gland secretions with different types of cellular debris and different skin microbiota^−^influenced metabolites [48,49,50]. However, the upper^−^back and armpit sites can be classified mainly as the eccrine and apocrine sweat glands, respectively [1], and were adopted in this study. Another weakness of this study was that a relatively small sample (six male university students) was recruited for the test. Even though the effect sizes (power in Table 2) for the analyses were close to or more than the criteria (i.e., 0.8) that suggested by Cohen [51], this small sample definitely alters the robustness of the results. In addition, the fitness of participants was not fully assessed. This may influence the homogeneity within the group of the exercise intensity and should be examined for future investigation. These limitations would inevitably restrict the generalization of the results and should be considered before the results are widely applied.

## 5. Conclusions

Urea, Na^+^, and K^+^ secretions were significantly higher for the sweat produced by running than for that produced by a hot environment. Similar results were also observed in the comparison between the apocrine and eccrine sweat glands, respectively. By contrast, the secretion of Cl^−^ revealed nonsignificant effects on various sweating methods and sweat glands. Changes in the metabolic rate incurred during exercise may have caused the higher excretion rates during running than during inactive overheating. Differences between apocrine gland secretion by pinching off outer cell parts and eccrine secretion from the cell may have resulted in the higher urea and ion secretions from the apocrine glands. The findings of this study can serve as a reference for identifying the metabolisms of various sweat^−^gland types under different sweating conditions as well as for establishing recommended personal^−^hygiene habits.

## Figures and Tables

**Table 1:** Age, physical characteristics, and exercise habit of six male young participants.

Items	Mean	Standard Deviation	Range
Age (years)	22.2	0.4	22.0–23.4
Stature (cm)	171.9	7.8	161.5–179.1
Body weight (kg)	68.9	8.6	60.5–81.3
Resting heart rate (beats/min)	73.2	3.6	67–81
Maximal peak expiratory flow (*l*)	9.4	0.6	8.9–10.3
Exercise frequency (times/week)	2.8	1.1	2–4

**Table 2:** Summary of ANOVA results for the composition of sweat excretions.

	Compositions	DF	SS	MS	F	Significance	Power
Sweating condition	Urea	1	2109	2109	13.08	*P* < 0.005	0.930
	Na^+^	1	1013937	1013937	14.88	*P* < 0.001	0.956
	Cl^−^	1	1193942	1193942	0.59	*P* = 0.452	0.114
	K^+^	1	1920438	1920438	27.07	*P* < 0.001	0.999
Sweat gland	Urea	1	1926	1926	11.95	*P* < 0.005	0.908
	Na^+^	1	450182	450182	6.61	*P* < 0.05	0.786
	Cl^−^	1	6073222	6073222	2.99	*P* = 0.990	0.377
	K^+^	1	365313	365313	5.15	*P* < 0.05	0.714
Sweating condition × Sweat gland	Urea	1	376	376	2.33	*P* = 0.142	0.307
	Na^+^	1	87483	87483	1.28	*P =* 0.271	0.190
	Cl^−^	1	1200195	1200195	0.59	*P* = 0.450	0.114
	K^+^	1	206276	206276	2.91	*P* = 0.104	0.368

*Notes:* DF—Degree of freedom; SS—Sum of square; MS—Mean square; F—F value; power, Effect size.

## References

[1] Wilke K., Martin A., Terstegen L., Biel S.S. (2007). A short history of sweat gland biology. Int. J. Cosmet. Sci..

[2] Meyer F., Laitano O., Bar-Or O., McDougall D., Heigenhauser G.J.F. (2007). Effect of age and gender on sweat lactate and ammonia concentrations during exercise in the heat. Braz. J. Med. Biol. Res..

[3] Garden J.W. (1966). Plasma and sweat histamine concentrations after heat exposure and physical exercise. J. Appl. Physiol..

[4] Fellmann N., Labbe A., Gachon A.M., Coudert J. (1985). Thermal sweat lactate in cystic fibrosis and in normal children. Eur. J. Appl. Physiol..

[5] Åstrand P.O., Rodahl K. (1986). Textbook of Work Physiology.

[6] Huang C.T., Chen M.L., Huang L.L., Mao I.F. (2002). Uric acid and urea in human sweat. Chin. J. Physiol..

[7] Cone E.J. (1997). New developments in biological measures of drug prevalence. NIDA Res. Monogr..

[8] Dutkiewicz E.P., Lin J.D., Tseng T.W., Wang Y.S., Urban P.L. (2014). Hydrogel micropatches for sampling and profiling skin metabolites. Anal. Chem..

[9] Schiefferdecker P. (1922). The skin glands of humans and mammals and their biological and racial anatomical significance and the muscularis sexualis. Zoologica.

[10] Sato K., Sato F. (1987). Sweat secretion by human axillary apoeccrine sweat gland in vitro. Am. J. Physiol..

[11] Sato K., Kang W.H., Saga K., Sato K.T. (1989). Biology of sweat glands and their disorders. I. Normal sweat gland function. J. Am. Acad. Dermatol..

[12] Morgan R.M., Patterson M.J., Nimmo M.A. (2004). Acute effects of dehydration on sweat composition in men during prolonged exercise in the heat. Acta. Physiol..

[13] Baker L.B., Barnes K.A., Anderson M.L., Passe D.H., Stofan J.R. (2016). Normative data for regional sweat sodium concentration and whole-body sweating rate in athletes. J. Sports Sci..

[14] Verde T., Shephard R., Corey P., Moore R. (1982). Sweat composition in exercise and in heat. J. Appl. Physiol..

[15] Dill D.B., Hall F.G., Van Beaumont W. (1966). Sweat chloride concentration: Sweat rate, metabolic rate, skin temperature, and age. J. Appl. Physiol..

[16] Vairo D., Bruzzese L., Marlinge M., Fuster L., Adjriou N., Kipson N., Brunet P., Cautela J., Jammes Y., Mottola G. (2017). Towards addressing the body electrolyte environment via sweat analysis: Pilocarpine iontophoresis supports assessment of plasma potassium concentration. Sci. Rep..

[17] Berenson G.S., Burch G.E. (1952). The response of patients with congestive heart failure to a rapid elevation in atmospheric temperature and humidity. Am. J. Med. Sci..

[18] Consolazio C.F., Matoush L.O., Nelson R.A., Hackler L.R., Preston E.E. (1962). Relationship between calcium in sweat, calcium balance, and calcium requirements. J. Nutr..

[19] Hussain J.N., Mantri N., Cohen M.M. (2017). Working up a good sweat–the challenges of standardising sweat collection for metabolomics analysis. Clin. Biochem. Rev..

[20] Schwartz S. 9 Showering Mistakes That Can Actually Hurt You. StethNews, Updated April 14, 2015. www.huffpost.com/entry/showering-mistakes-_n_7065204.

[21] De Bruyne G., Aerts J.M., Vander Sloten J., Goffin J., Verpoest I., Berckmans D. (2010). Transient sweat response of the human head during cycling. Int. J. Ind. Ergon..

[22] Jadoon S., Karim S., Akram M.R., Kalsoom Khan A., Zia M.A., Siddiqi A.R., Murtaza G. (2015). Recent developments in sweat analysis and its applications. Int. J. Anal. Chem..

[23] Baker L.B. (2017). Sweating rate and sweat sodium concentration in athletes: A review of methodology and intra/interindividual variability. Sports Med..

[24] De Giovanni N., Fucci N. (2013). The current status of sweat testing for drugs of abuse: A review. Curr. Med. Chem..

[25] Harker M., Coulson H., Fairweather I., Taylor D., Daykin C.A. (2006). Study of metabolite composition of eccrine sweat from healthy male and female human subjects by 1 h NMR spectroscopy. Metabolomics.

[26] Kutyshenko V.P., Molchanov M., Beskaravayny P., Uversky V.N., Timchenko M.A. (2011). Analyzing and mapping sweat metabolomics by high-resolution NMR spectroscopy. PLoS ONE.

[27] Genuis S.J., Beesoon S., Lobo R.A., Birkholz D. (2012). Human elimination of phthalate compounds: Blood, urine, and sweat (BUS) study. Sci. World J..

[28] Mark H., Harding C.R. (2013). Amino acid composition, including key derivatives of eccrine sweat: Potential biomarkers of certain atopic skin conditions. Int. J. Cosmet. Sci..

[29] Sheng J., Qiu W., Xu B., Xu H., Tang C. (2016). Monitoring of heavy metal levels in the major rivers and in residents’ blood in zhenjiang city, china, and assessment of heavy metal elimination via urine and sweat in humans. Environ. Sci. Pollut. Res..

[30] Tang. S., Yu X., Wu C. (2016). Comparison of the levels of five heavy metals in human urine and sweat after strenuous exercise by ICP-MS. J. Appl. Math. Phys..

[31] Ely M.R., Kenefick R.W., Cheuvront S.N., Chinevere T.D., Lacher C.P., Lukaski H.C., Montain S.J. (2011). Surface contamination artificially elevates initial sweat mineral concentrations. J. Appl. Physiol..

[32] Mackintosh C.A., Lidwell O.M., Towers A.G., Marples R.R. (1978). The dimensions of skin fragments dispersed into the air during activity. Epidemiol. Infect..

[33] Groscurth P. (2002). Anatomy of Sweat Glands. Hyperhidrosis and Botulinum Toxin in Dermatology.

[34] Al-Tamer Y.Y., Hadi E.A. (1997). Sweat urea, uric acid and creatinine concentrations in uraemic patients. Urol. Res..

[35] Taylor R.P., Polliack A.A., Bader D.L. (1994). The analysis of metabolites in human sweat: Analytical methods and potential application to investigation of pressure ischaemia of soft tissues. Ann. Clin. Biochem..

[36] Hoshi A., Watanabe H., Kobayashi M., Chiba M., Inaba Y., Kimura N., Ito T. (2001). Concentrations of trace elements in sweat during sauna bathing. Tohoku J. Exp. Med..

[37] Taylor N.A., Machado-Moreira C.A. (2013). Regional variations in transepidermal water loss, eccrine sweat gland density, sweat secretion rates and electrolyte composition in resting and exercising humans. Extreme Physiol. Med..

[38] Appenzeller B.M., Schummer C., Rodrigues S.B., Wennig R. (2007). Determination of the volume of sweat accumulated in a sweatpatch using sodium and potassium as internal reference. J. Chromatogr. B.

[39] Sato K. (1977). The physiology, pharmacology, and biochemistry of the eccrine sweat gland. Rev. Physiol. Biochem. Pharmacol..

[40] Weschler L.B. (2008). Sweat electrolyte concentrations obtained from within occlusive coverings are falsely high because sweat itself leaches skin electrolytes. J. Appl. Physiol..

[41] Quinton P.M. (1981). Effects of some ion transport inhibitors on secretion and reabsorption in intact and perfused single human sweat glands. Pflügers Archiv.

[42] Sato K., Gisolfi C.V., Lamb D.R., Nadel E.R. (1993). The mechanism of eccrine sweat secretion. Perspectives in Exercise Science and Sports Medicine. Exercise, Heat, and Thermoregulation.

[43] Reddy M.M., Quinton P.M. (1994). Rapid regulation of electrolyte absorption in sweat duct. J. Membr. Biol..

[44] Huxley H. (1969). The mechanism of muscular contraction. Science.

[45] Costill D.L. (1984). Water and electrolyte requirements during exercise. Clin. Sports Med..

[46] Buono M.J., Claros R., DeBoer T., Wong J. (2008). Na+ secretion rate increases proportionally more than the Na+ reabsorption rate with increases in sweat rate. J. Appl. Physiol..

[47] Fukumoto T., Tanaka T., Fujioka H., Yoshihara S., Ochi T., Kuroiwa A. (1988). Differences in composition of sweat induced by thermal exposure and by running exercise. Clin. Cardiol..

[48] Belkaid Y., Segre J.A. (2014). Dialogue between skin microbiota and immunity. Science.

[49] Delgado-Povedano M.M., Calderón-Santiago M., de Castro M.L., Priego-Capote F. (2018). Metabolomics analysis of human sweat collected after moderate exercise. Talanta.

[50] Fyhrquist N., Salava A., Auvinen P., Lauerma A. (2016). Skin biomes. Curr. Allergy Asthma Rep..

[51] Cohen J. (1988). Statistical Power Analysis for the Behavioral Sciences.

